# Treatment of Patellofemoral Pain Syndrome with Dielectric Radiofrequency Diathermy: A Preliminary Single-Group Study with Six-Month Follow-Up

**DOI:** 10.3390/medicina57050429

**Published:** 2021-04-28

**Authors:** Manuel Albornoz-Cabello, Cristo Jesús Barrios-Quinta, Isabel Escobio-Prieto, Raquel Sobrino-Sánchez, Alfonso Javier Ibáñez-Vera, Luis Espejo-Antúnez

**Affiliations:** 1 Department of Physiotherapy, Faculty of Nursing, Physiotherapy and Podiatry, University of Sevilla, 41009 Sevilla, Spain; malbornoz@us.es; 2 Physiotherapy Unit, Andalusian Health Service, 41005 Sevilla, Spain; cristo.barrios@gmail.com; 3 Department of Physiotherapy, Universitary School of Osuna, 41640 Sevilla, Spain; raquelss@euosuna.org; 4 Health Sciences Department, University of Jaen, 23071 Jaen, Spain; ajibanez@ujaen.es; 5 Department of Medical-Surgical Therapy, Medicine Faculty, University of Extremadura, 06071 Badajoz, Spain; luisea@unex.es

**Keywords:** radiofrequency, patellofemoral pain syndrome, health, quality of life, physical therapy

## Abstract

*Background and Objectives*: Notwithstanding patellofemoral pain syndrome (PFPS) being one of the most common causes of pain in the front of the knee in outpatients, few studies have shown the effects of radiofrequency on knee pain and function in this population. The aim of the present study was to determine whether outpatients diagnosed with PFPS obtained improvement in pain and function after treatment by dynamic application of monopolar dielectric diathermy by emission of radiofrequency (MDR). *Materials and Methods*: An experimental study was conducted with 27 subjects with PFPS. Subjects were treated with 10 sessions of MDR in dynamic application. The visual analogue scale (VAS), the Kujala scale, the DN4 questionnaire, the lower extremity function scale (LEFS), the range of movement (ROM) in knee flexion and extension and the daily drug intake were measured pre- and post-intervention and at the time of the follow-up (six months). *Results*: Statistically significant differences were found in pain perception (VAS: F_1,26_ = 92.43, *p* < 0.000, ŋ^2^ = 0.78 and DN4: F_1.26_ = 124.15, *p* < 0.000, ŋ^2^ = 0.82), as well as improvements in functionality (LEFS: F_1.26_ = 72.42, *p* < 0.000, ŋ^2^ = 0.74 and Kujala: F_1.26_ = 40.37, *p* < 0.000, ŋ^2^ = 0.61]) and in ROM (Flexion: F_1.26_ = 63.15, *p* < 0.000, ŋ^2^ = 0.71). No statistically significant changes in drug intake were found. *Conclusions*: The present study shows that the dynamic application of MDR seems effective in reducing pain and increasing functionality and knee flexion in patients with PFPS, after a follow-up of six months.

## 1. Introduction

Patellofemoral pain syndrome (PFPS) is a health condition of maximum topicality [1,2,3]. It is a common musculoskeletal dysfunction, which tends to be chronic and presents as pain in the front of the knee [4]. It tends to occur more commonly in adolescents and young adults, although it does affect all population groups [5]. PFPS has usually been associated with obesity and knee osteoarthritis, but a recent study observed the inconsistency of this relationship [6]. Activities such as walking upstairs of downstairs, running, squatting or even sitting for a long time, seem to aggravate the symptoms, which could be related to the fact that compression forces in the patellofemoral joint are increased in these movements [7]. PFPS is still one of the most common and challenging muscle-skeletal dysfunctions for physiotherapists and sport medicine professionals [8].

Although the pain associated with PFPS is very characteristic, the causes of pain are as of yet unknown, traditionally being linked to cartilage damage. However, joint cartilage is known to have no nerve supply [9]. As there is a wide variety of pathologies that can present similar signs and symptoms as PFPS, the term is used to describe all front of the knee pain [10].

There is a large list of therapeutic approaches for the treatment of PFPS, likely due to a lack of understanding with regard to the etiology of the pain and dysfunction intrinsic to this condition. Physiotherapy is the most common approach within non-surgery-based treatments, particularly at the beginning of the pathology [8], including strengthening the vastus medialis of the quadriceps to improve the active stability of the patella in the femoral trochlea, patellar realignment taping, knee and hip muscle stretching, manual therapy and therapeutic exercises [11,12,13]. However, evidence supporting the efficacy of these clinical practices is still scarce, despite the fact that these treatments appear to be based on sound clinical reasoning [10].

Hence, to accomplish the aim of the present study, among all the physiotherapy tools available, the electrotherapy treatment based on the application of monopolar dielectric diathermy by emission of radiofrequency (MDR) was selected. Few studies exist concerning its application in PFPS, despite being a frequently used physiotherapy technique. Non-statistically significant results were obtained in a 2012 study by Verma and Krishnan comparing the application of short-wave and strengthening vastus medialis exercises to McConell patellar taping and the same strengthening exercises [14]. In addition, a recent study by Albornoz-Cabello et al., (2020) concluded that the addition of MDR treatment in addition to home knee therapeutic exercises obtained greater results for pain than home exercises alone [15]. In this line, it remains unknown whether the MDR treatment alone would be effective in pain reduction.

For all the above reasons, the aim of the present study was to assess the effects of MDR diathermy, after the follow-up of six months, in pain measured with VAS, in the neuropathic component of pain measured with DN4 [16,17] and in knee function assessed by range of motion (ROM), the Kujala score [18] and the lower extremity functional scale (LEFS) [19].

## 2. Materials and Methods

### 2.1. Study Design

The design of this experimental study was a prospective uncontrolled trial. The study period was from May to December 2019. The research protocol was approved by the Andalusian Health Service Research Ethics Committee (Reference: CEI 1696-N-17, date 1/29/2018). Before inclusion, all participants signed an informed consent form agreeing to participate after being duly informed about the study and their rights.

### 2.2. Participants

Patients over 18 years of age with PFPS were referred by a primary healthcare physician in the La Rinconada de Sevilla district (Seville, Spain). Only chronic patients who had undergone pharmacologic analgesic treatment and home exercise prescription, with a pain score of 30 mm and over in VAS and below 45 in the psychological apprehension scale (PPAS) [20], were invited to take part. PPAS is an easy-to-use, reliable, useful and validated tool in order to assess subjects’ apprehension of receiving electrical stimulation therapy [21]. Exclusion criteria included all cases where the use of MDR was contraindicated (fever, active process of tuberculosis, pregnancy, infected wounds, osteomyelitis, tumors, presence of implanted electronic devices such as pacemakers, rheumatoid arthritis, thrombophlebitis or deep venous thrombosis) [22,23,24,25], and/or cases having received at any time treatment with corticoid or hyaluronic acid injections, cognitive or communicative impairment or being involved in a medical-legal dispute at the time. To avoid possible significant changes in the study, patients were asked to maintain their usual physical activity and treatments throughout the study, but they were allowed to use paracetamol; however, drug intake data was recorded to monitor any possible influence on final measurements.

### 2.3. Study Protocol

Measurements were collected by a blinded researcher at baseline, post-intervention and at six months of follow-up after the last treatment. The treatment protocol consisted of a three-week intervention: the first week consisted of five treatment sessions (Monday to Friday); the second, three sessions (Monday, Wednesday, and Friday); and the third, only two treatment sessions (Monday and Thursday). Each session took 12 minutes and was performed by a physiotherapist with 15 years of experience using MDR. The whole study lasted for six months, which included three weeks of intervention and another six months of follow-up period without intervention.

### 2.4. Measurements

To assess the maximum pain experienced by the patients included in the study in the previous 24 h, the were asked to use VAS, which normally consists of a horizontal line, whereby a score of 0 mm would mean “absence of pain” and 100 mm would mean “the most insufferable pain” [26]. The minimal clinically important differences for VAS were determined as the reduction of 15–20% [27] or of 14 mm in VAS [28] after the intervention and follow-up. Pain intensity was described by the patient, who pointed to a number on the line. In order to assess neuropathic pain, the tool used was the DN4 questionnaire, which scores from 0 to 10 [16,17]. The lower limb functionality was evaluated with the Kujala score [18] and the LEFS [19]. Finally, a conventional two-arm goniometer was used to passively determine knee ROM in flexion and extension.

### 2.5. Interventions

**Figure 1:**
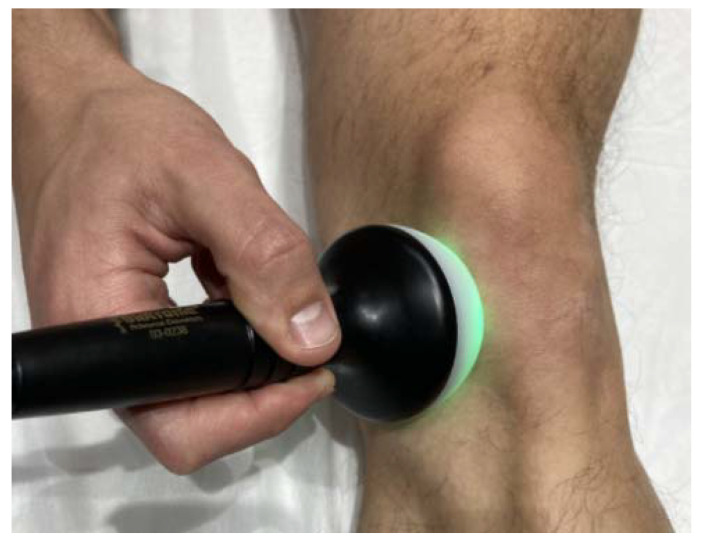
Application of MDR.

MDR treatment was applied using ABD Modular^®^, a monopolar dielectric diathermy radiofrequency device (Biotronic Advance Develops^®^, Granada, Spain). The applicator of this type of device must be used with dielectric substances instead of conductive ones, which is the most important differential point between the monopolar dielectric transmission and the bipolar capacitive-resistive one [22]. The dielectric substance minimizes heating on tissue surfaces, thereby focusing energy on depth [22]. Participants received MDR treatment with a dynamic pulsed emission of 840 kHz and 30 V, with continuous rotary movement covering all the front surface of the knee from lateral to medial aspect. Five milliliters of almond oil were employed as the dielectric substance so as to improve gliding throughout the 12-min MDR application [22,23] (Figure 1).

### 2.6. Statistical Analysis

To calculate sample size, we relied on the detection of an improvement of 20% in self-perceived pain intensity [27] Considering a repeated measures ANOVA within factors, an alpha value of 0.05, a desired power of 80% and a small effect size (f = 0.26), 26 participants were required for treatment (G * Power, version 3.1.2, Kiel University, Kiel, Germany).

The statistical analysis of the data was performed with PASW advanced statistics (SPSS Inc. Chicago, IL, USA), version 24.0. Results were reported as mean (standard deviation) and confidence intervals (CI 95%). The normal distribution of the studied variables was verified by the Shapiro–Wilk test, after the appropriate descriptive analysis. Besides, the Levene test was used to assess the homogeneity of variances. To assess linearity, bivariate dispersion graphics of residual values were observed from the expected values. Differences in measurements were determined by analysis of variance of repeated measures within factors (ANOVA) to evaluate time interactions, including the effect of time (baseline, three weeks after treatment and six months after treatment). Eta square (ŋ^2^) was chosen to calculate the effect size, considering it small when 0.01 ≤ ŋ^2^ ≤ 0.06, moderate when 0.06 ≤ ŋ^2^ > 0.14 and large when ŋ^2^ > 0.14. Statistical significance was determined at *p* < 0.05.

## 3. Results

### Description of the Sample

**Figure 2:**
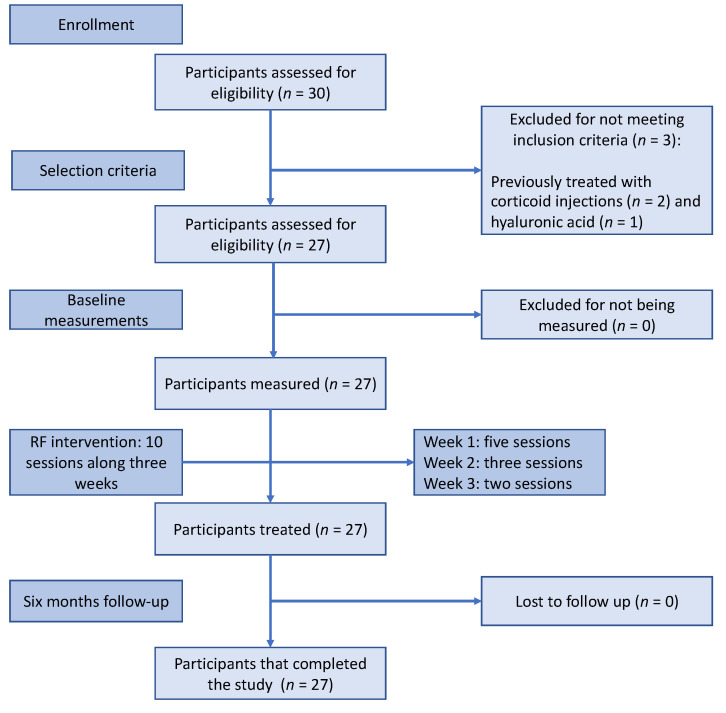
Flow diagram of participants.

A final amount of 30 adult subjects (aged between 19 and 65) were selected for this experimental study. Although finally, 27 participants were enrolled in the study (*n* = 27) (Figure 2), comprising 10 men and 17 women with a mean age of 48 ± 16.68 years. Regarding the patients’ knees treated, 14 were right (52%), while the remaining 13 were left (48%).

The baseline, post-treatment and follow-up demographic characteristics (body mass index, fat mass, metabolic age, BMR) are shown in Table 1.

The clinical outcomes (self-perceived pain measured with VAS and DN4, functionality measured with LEFS and Kujala test and flexion and extension ROM) are shown in Table 2.

Considering an improvement over the 20% in VAS as responding to the treatment, from the 27 participants, 14 (52%) improved after the three weeks of treatment, while 25 (93%) registered almost that improvement at the follow-up appointment.

**Figure 3:**
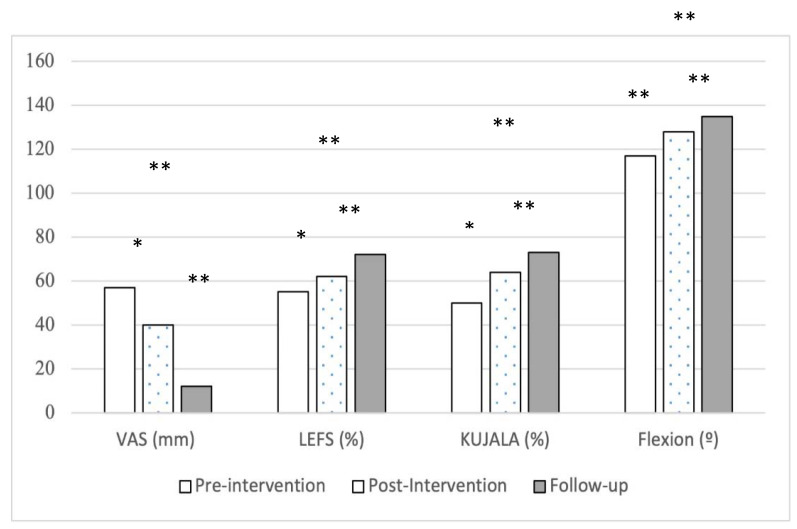
Time per group interaction for VAS, LEFS, Kujala and Flexion range of movement. *: *p* < 0.05; **: *p* < 0.001.

Statistically significant differences were found in pain self-perception (VAS F_1,26_ = 92.43, *p*< 0.000, ŋ^2^ = 0.78 and DN4: F_1,26_ = 124.15, *p*< 0.000, ŋ^2^ = 0.82), in knee disability (LEFS: F_1,26_ = 72.42, *p*< 0.000, ŋ^2^ = 0.74 and Kujala: F_1.26_ = 40.37, *p*< 0.000, ŋ^2^ = 0.61) and in range of movement (Flexion: F_1.26_ = 63.15, *p*< 0.000, ŋ^2^ = 0.71) (Figure 3). No statistically differences were found between the before treatment, after treatment and follow-up measurements regarding the use of basic analgesic drugs; in fact, none of the participants referred having used paracetamol during the study. Concerning possible side effects of the treatment, it must be considered that none were reported or observed.

## 4. Discussion

The aim of this study was to investigate the clinical effects of the short- and long-term use of a popular electrotherapy technique such as MDR in the treatment of PFPS. Although there are many studies concerning PFPS, no strong evidence exists to support any specific treatment. As a non-surgical intervention, physiotherapy is widely used to treat PFPS: taping techniques and knee exercises seem to be the most effective treatment [10,12,13,14], but differing results have been found upon comparing studies [8].

The effects that diathermy devices based on emissions of radiofrequency have on knee pain have only been studied by few authors. In a recent study by Albornoz-Cabello et al., (2020), the addition of MDR to knee home exercises obtained greater pain improvements than exercise alone in participants with patellofemoral pain syndrome [15]. In another study conducted by Albayrak et al., (2017), better results were obtained for the neuropathic pain component of total knee arthroplasty patients in the group using radiofrequency added to transcutaneous electrical nerve simulation (TENS) than in the one using only TENS [29]. Meanwhile, the present study revealed significant improvements in both pain and neuropathic pain components, leading to the implication that a non-invasive application directly over the front of the knee is more effective than the invasive one used by Albayrak et al. [29]. Further, the type of device used in the present study was monopolar dielectric as opposed to the bipolar resistive one used by Albayrak et al., which could also represent a differential point. In our study, 13 participants presented a neuropathic pain component at baseline according to Pérez et al. [17], with five of them improving after the treatment and the remaining eight at the follow-up. This excellent result obtained in our study in regard to the neuropathic pain component must be considered with caution, as we think that such an important improvement is unexpected in such a complex pathology, pointing to a possible misdiagnose. Thus, further studies are needed to explore the effects of MDR in neuropathic pain.

Considering the results of Albornoz-Cabello et al., (2020) [15], the similar improvements in pain registered in this study point to the fact that MDR treatment by itself is effective in pain reduction, although exercise increases the therapeutic effect. Moreover, our study revealed that effects of MDR last for almost six months, a point that was unsolved by Albornoz-Cabello et al., as that study did not perform a follow-up.

Another study carried out by Verma et al., (2012) revealed slightly better results for the treatment of PFPS with McConell taping plus exercises compared to short-wave diathermy plus exercises [14]. It is important to note that it is not possible to establish an effective comparison between the Verma study and ours (between short-wave diathermy and MDR treatments), for two main reasons: 1) the type of diathermy (frequency vs. short-wave) [30] and 2) the depth of treatment [22]; moreover, even the consistency of the results is limited by the small sample used by Verma (*n* = 20).

As noted above, the most studied treatments for PFPS are taping and exercises. It can be seen from McConell’s study in patients with PFPS that taping for patellar alignment plus knee motor control and eccentric exercises represents a good a long-term solution, showing improvements in both pain and knee function [13]. However, the latest systematic review from Logan et al. on taping techniques for PFPS concluded that taping increases effectiveness only when used in combination with exercises, yielding poor clinical results by itself [10]. In the same way, although exercise by itself was also found to produce poorer results than taping plus exercises, the role of exercise might be suggested to be more important than taping [10], in concordance with the recent study of Albornoz et al., in which a program with therapeutic exercises was effective at reducing pain and disability in patients with PFPS [31]. According to the results of our study, a combination incorporating taping, knee exercises, and MDR could yield impressive results for PFPS management, encouraging future studies to follow this approach.

Thus, regarding the previous information and the data generated by the present study, it can be considered that MDR could represent an effective treatment in managing knee functionality, range of motion and pain. However, the complex physiopathology of PFPS requires more studies in order to confirm whether or not improved clinical benefits can be achieved by combining this treatment with taping and knee exercises. Considering previous studies in patients with musculoskeletal conditions, such as supraspinatus tendinopathy [32] and lower back pain [33], using distinct types of diathermy by emission of radiofrequency, we can consider this technique to be valid in the treatment of musculoskeletal pain and joint function.

The present study has several limitations. First of all, a control group would be required to assess the possible interaction of time in outcomes. Secondly, the localization reduces the generalizability of the results; thus, future studies are needed with a multicenter design. Nevertheless, although participants were asked to avoid any change in their usual physical activity or treatment during the study, we cannot assure that all of them followed this. Further studies should consider higher cut-off values for clinical success and comparisons with other validated treatments.

## 5. Conclusions

In the present study, participants with patellofemoral pain syndrome treated with monopolar dielectric diathermy by emission of radiofrequency obtained improvements in pain, the neuropathic pain component and functionality in the short term and at the six-month follow-up. Therefore, the application of MDR by a physiotherapist could be effective in pain reduction and function improvement. Further studies are needed to assume that improvements were due to intervention and not to time.

## Figures and Tables

**Table 1:** Pre-intervention, post-intervention and follow-up mean score changes of characteristics of the sample.

	Pre-Intervention	Post-Intervention	Follow-Up	Pre-/Post-Differences	Post-/Follow-Up Differences	Pre-/Follow-Up Differences
BMI	27.3 ± 4.1	27.5 ± 3.8	27.2 ± 3.5	0.2 (−0.9/0.5)	0.3 (−0.01/0.7)	0.1 (−0.7/1.0)
Fat mass (%)	31.9 ± 8.7	30.4 ± 10.5	29.7 ± 10.5	1.5 (−2.6/5.6)	0.7 (0.2/1.1)	2.2 (−1.9/6.4)
Metabolic age (year)	49 ± 16.7	48 ± 17.6	48 ± 17.7	1 (−5/6)	0 (0/1)	1 (−4/7)
BMR (KJ)	6425 ± 1502.9	6622 ± 1440.8	6767 ± 1498.1	196 (−634/240)	144 (13/302)	341 (135/818)

Data are reported as mean ± SD or (95% confidence level); BMI: body mass index.

**Table 2:** Pre-intervention, post-intervention and follow-up mean score changes of knee pain and lower extremity function.

	Pre-Interv	Post-Interv	F-U	Pre-/Post-Differences	% ∆ (d)	Post-/F-U Differences	% ∆ (d)	Pre-/F-UDifferences	% ∆ (d)
VAS (mm)	57 ± 20.3	40 ± 33.3	12 ± 15.9	17 (4.6/28) *	−30 (0.62)	28 (15/42) **	−70 (1.07)	45 (35/55) **	−79 (2.46)
DN4 (0–10)	4.4 ± 2.1	2.6 ± 2.5	0.4 ± 0.8	1.8 (0.7/3) *	−41 (0.78)	2.2 (1.2/3.2) **	−85 (1.18)	4.0 (3.3/4.8) **	−91 (2.52)
LEFS (%)	55 ± 20.6	62 ± 19.7	72 ± 17.6	7 (1/12) *	13 (0.35)	10 (6/13) **	16 (0.53)	17 (13/21) **	31 (0.89)
Kujala (%)	50 ± 23.5	64 ± 20.7	73 ± 17.4	14 (5/22) *	28 (0.63)	9 (5/13) **	14 (0.47)	23 (15/30) **	46 (1.11)
Flex (º)	117 ± 12.4	128 ± 7.8	135 ± 6.3	11 (6/15) **	9 (1.06)	7 (4/9) **	5 (0.99)	18 (13/22) **	15 (1.83)
Ext (º)	1 ± 2.1	0 ± 1.3	0 ± 0	1 (0/1)	-	0 (0/1)	-	0 (0/1)	-

Data are reported as mean ± SD or (95% confidence level) and the difference (∆, in%) (d de Cohen); * Indicates statistically significant pre-post and follow-up differences (*p* < 0.05); ** Indicates statistically significant pre-post and follow-up differences (*p* < 0.001). Flex: knee flexion range of movement; Ext: extension knee range of movement; Interv: intervention; F-U: follow-up.

## Data Availability

Data available under request to the first author of the study.
